# Levothyroxine personalized treatment: is it still a dream?

**DOI:** 10.3389/fendo.2023.1334292

**Published:** 2024-01-08

**Authors:** Carlo Cappelli, Elisa Gatta, Salvatore Ippolito

**Affiliations:** ^1^ Department of Clinical and Experimental Sciences, SSD Endocrinologia, University of Brescia, ASST Spedali Civili of Brescia, Brescia, Italy; ^2^ Consulcesi Homnya, Head of Omnichannel Strategy & Project Management, Rome, Italy

**Keywords:** tailored pharmaceuticals, precision pharmaceuticals, personalized drug formulations, targeted drug delivery, levothyroxine, hypothyroidism treatment, L-T4 personalized treatment

## Abstract

Levothyroxine is a milestone in the treatment of all causes of hypothyroidism. From 19th century till today, Levothyroxine experienced a great advancement, from hypodermic injections of an extract of the thyroid gland of a sheep to novel formulations, known to circumvent malabsorption issue. However, the rate of patients on suboptimal therapy is still high. Current Guidelines are clear, daily Levothyroxine dosage should be calculated based on body weight. However, we are still far away from the possibility to administer the right dosage to the right patient, for several reasons. We retrace the history of treatment with levothyroxine, pointing out strengths and weaknesses of different formulations, with particular attention to what keeps us away from tailored therapy. In the age of digitalization, the pharmaceutical industry has been giving rising importance to Digital therapeutics, that are known to be effective in reaching target therapies. By combining current knowledge of hypothyroidism therapy with cutting-edge technology, we also hypothesized what could be the future strategies to be developed in this field.

## Introduction

Levothyroxine (L-T4) is a milestone in the treatment of all causes of hypothyroidism, being the second most common medication dispensed in the United States in the last few years (1, 2). The first documented effective myxedema treatment with hypodermic injections of an extract of the thyroid gland of a sheep dates back to the late 19^th^ century (3). In 1926 thyroxine was synthetized (4), although L-T4 was commercially marketed in 1955. However, tablets of desiccated thyroid extract, containing both thyroxine and triiodothyronine, remained the primary therapy until 1970s (5). L-T4 tablets represented the unique available therapy for over 30 years, until novel formulations (liquid oral solution and softgel capsules) were produced and distributed in 2000s (6). Even if L-T4 treatment appears to be easy to manage (7), it has a narrow therapeutic index (8) so much so that almost 50% of treated patients show abnormality of thyroid hormone profile after one year of treatment (9), potentially leading to iatrogenic complications or hypothyroidism symptoms (10). Many causes could be responsible of it, among them the lack of dosages that can enable precision therapy. We retraced the history of treatment with L-T4, pointing out strengths and weaknesses of different formulations, up to dream a personalized L-T4 treatment.

## The history

The thyroid gland’s significance was established in the 19^th^ century, however the discovery of thyroid hormones dates back to the 20^th^ century, and the treatment of hypothyroidism has been refined over the last century. In 1884, Moritz Schiff showed in animal models that the clinical effects of hypothyroidism following thyroidectomy significantly diminish when additional thyroid glands from an animal of the same species are introduced and implanted in the abdominal cavity beforehand (3). Some year later Bettencourt and Serrano apply this result in human, unfortunately the patient died three days after for titania (11). In the same years, George Murray reported the first case of myxedema treated successfully with hypodermic injections of an extract of the thyroid gland of a sheep (12, 13). In 1914, Edward Calvin Kendall and Edward L. Adkins extracted the hormone, namely thyroxine, from animal-derived desiccated thyroid glands. However, they did not determine its chemical structure (14). In 1926, Barger and Harington succeeded in synthesizing thyroxine (4). Although thyroxine was commercially marketed in 1955, tablets of desiccated thyroid extract, containing both thyroxine (T4) and triiodothyronine (T3) were commonly used until 1970s (5). Desiccated thyroid showed large variability in hormone content from batch to batch making difficult to achieve the consistent dosage and to maintain stable TSH levels (15, 16), and T3 concentrations could be fluctuating and often elevated, leading to the symptoms of hyperthyroidism (2, 17, 18). In light of these, in 1970s, the British endocrinologists felt compelled to warn against the use of desiccated thyroid (19). Twenty-five years later, the American Thyroid Association (ATA) Guidelines recommended against biological and synthetic thyroid hormone preparations containing T4 and T3 (20). Even today, the Guidelines of the most authoritative association do not recommend the use of desiccated thyroid extract, and they consider L-T4 monotherapy the preferred treatment (2, 21–23). Thus, for 50 years now, the consumption of L-T4 grew up worldwide.

It is well known that approximately 60- 90% of the L-T4 is absorbed in the jejunum and ileum within three hours of ingestion (24), and absorption is maximal when it is taken on an empty stomach. Once absorbed, T3 is obtained through deiodination reactions of L-T4 by deiodinase enzymes in peripheral tissues (25). Liver is the major site of deiodination but also kidney plays a significant role in the peripheral metabolism of thyroxine (26), however multiple tissues are capable of T4 deiodination to T3, then restoring the body’s T3 reservoir (27–33). The L-T4 half-life after L-T4 oral administration ranges from 6.2 to 7.5 days (34).

Indeed, the acid gastric pH is essential to dissolve the tablet, removing sodium ion and converting L-T4 into a lipophilic molecule (35). All the conditions altering acid gastric pH are in fact the main cause of serum TSH instability (9): hydrophilic sodium salt remains undissociated in hypochlorydic gastric conditions, thus less absorbed (36). For this reason, current Guidelines by a Task Force of the ATA recommend that L-T4 should be taken in a fasting state (2). In addition, many pathological conditions (i.e., autoimmune gastritis, *Helicobacter pylori* infection and bariatric surgery) and drugs (*i.e.*, proton pump inhibitors, ferrous sulphate, and calcium carbonate) reduce L-T4 absorption by alkalization of gastric pH or, in the case of some medications, the binding into insoluble complexes (37–41). In this view, ATA Guidelines suggest that L-T4 has to be taken away also from interfering drugs (2).

Hence, the need to provide an advanced therapy prompted pharmaceutical companies to develop new formulations: liquid oral solutions and softgel capsules (6). The first is composed only of L-T4 of variable concentrations and glycerin: no dissolution is therefore required thus immediately available for absorption (41). The latter is L-T4 dissolved in glycerin in an outer gelatin shell, providing protection from the variations of gastric pH and preventing the binding to other substances in the intestinal lumen (41). As theoretically expected, Yue et al. firstly demonstrated that liquid L-T4 reached systemic circulation faster than tablets, since dissolution is not needed before absorption starts (42). Going on, our group demonstrated that liquid L-T4 can be ingested directly at breakfast, with no significant difference of thyroid hormones profile (43, 44), improving patients’ compliance and quality of life (45, 46). Finally, liquid L-T4 formulations represent a milestone for patients diagnosed with condition that can somehow impair gastric acidity (39, 47–55) and moreover, they can be taken simultaneously with drugs known to interfere with L-T4 absorption (37, 43, 56–66). Few promising data are available about Softgel capsules (37). Recently a prospective study showed that soft gel capsule can circumvent malabsorption in presence of interfering drugs (64). However, more studies are needed to confirm these data.

Although there has been significant progress in the pharmacokinetics of levothyroxine, we are still far from personalized therapy. In fact, ATA Guidelines suggest a dosage of 1.6-1.8 μg/kg; higher doses of 2.0–2.1 μg/kg are required for patients on L-T4 suppressive therapy for differentiated thyroid cancer (2). Most available formulations come with predetermined dosages. Even if most recent formulations, such as Levotirsol® (IBSA Farmaceutici Italia), have been commercialized with a wide range of intermediate dosages, the possibility to administer to each patient the right dosage remains an unlikely scenario.

## Discussion

Even though the management of hypothyroidism is generally considered straightforward, almost half of patients showed TSH serum levels indicating over- or under-treatment (67, 68).

Maintaining TSH levels within the normal range adjusted for age and comorbidities is crucial to avoid deleterious effects. In fact, on one side, the risk for inpatient admissions and deaths due to cardiovascular disease, dysrhythmias, and osteoporotic fractures is higher for patients on L-T4 with suppressed TSH values, especially in elder people and postmenopausal women (69–71). On the other side, inadequate therapy is associated with dyslipidemia, atherosclerotic cardiovascular disease, and congestive heart failure, although likely to a less severe degree (72–74).

Many causes could explain the difficulty in maintaining TSH within normal ranges through the years, and among them the absence of a tailored L-T4 dosage has to be considered.

What should the gold standard be for a personalized L-T4 treatment? The answer is simple: the possibility to have a dosage of 1.6-2.1 μg/kg as recommended by American Thyroid Association guidelines (2), a steady absorption with a good compliance. Up to today, we have not had the possibility to give L-T4 dosage tailored for the patient’s weight. Indeed, liquid formulation as drops (Tirosint®, IBSA Farmaceutici Italia) or oral solution (Tifactor®, I.B.N. Savio Italia) permits to be close to a satisfactory personalized treatment. In fact, each milliliter of Tifactor® contains from 5 mcg to 20 mcg of L-T4, whereas each Tirosint® oral drop contains 3.75 mcg of L-T4, that is a good but not perfect dosage. However, this therapeutic strategy does not enhance patients’ compliance since counting drops is not practical, in particular for elder and partially sighted patients.

In the ever-evolving age of digitalization, the pharmaceutical industry has been giving rising importance to Digital therapeutics (DTx), driven by the purpose of transforming digital technologies into treatments (75). These systems deliver evidence-based therapeutic interventions to patients that are driven by a software that is programmed to prevent, manage, alleviate or treat a medical disorder or disease (76). Digital therapeutics can be used independently or in combination with medications, devices, or other therapies (77). What is more effective in reaching target therapy than a digital system? Undoubtedly, the most direct beneficiaries of DTx are patients themselves, by improving their experience, outcomes, and the coordination of their care; but patients aren’t the only ones benefitting (78). In fact, DTx improve the capability of clinicians to monitor treatment and to obtain information about care and patients’ response, using data sharing between patients and healthcare professionals (79). Moreover, also healthcare systems can profit, by targeting underserved areas in healthcare and reducing the burden on healthcare systems, by preventing hospital visits, improving self-management or providing therapy remotely (79).

## Future direction

Precision medicine, also known as personalized medicine, is an innovative approach for tailoring diseases that allows the selection of a treatment in order to provide the best result limiting or deleting side effects. In the last few years, the need to detect the right drug, with the right dosage at the right time to the right patient has increased the interest of pharmaceutical companies and researchers (80). This “revolution” has been spreading in health care, particularly in oncology, providing effective tailored therapeutic strategies based on epigenomic, proteomic, and genomic profiles of each patient (81–85). Simultaneously, the growing development of medical engineering and miniaturizing devices, increased patients’ compliance and quality of life. An anecdotical example is the artificial pancreas: a closed-loop insulin injection system that continuously monitors glucose levels and automatically adjusts insulin injection in real time (86). This permits a better quality of life for patients (87), reducing hypoglycemia (88), improving therapeutic target achievement (89), but above all, reducing overall mortality (90). As it is well demonstrated for insulin pumps, health technologies are cost-effective (91): in front of a high upfront investment, several economic benefits can be obtained, in particular healthcare systems can display a reduction of direct and indirect costs.

**Figure 1:**
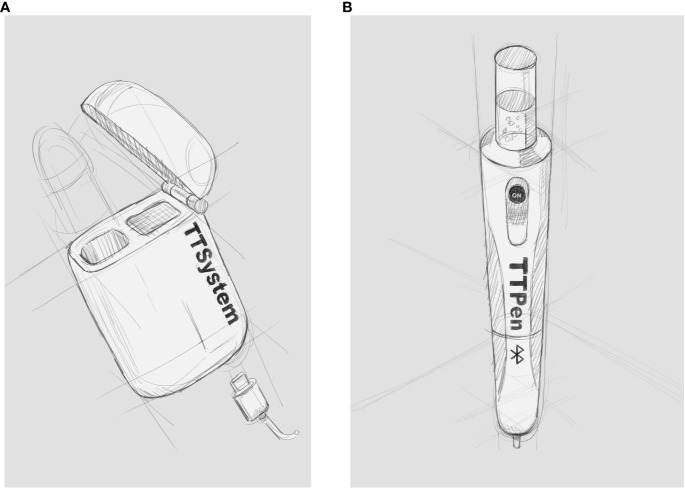
Graphical aspect of TTPen. Caption: **(A)** Charger of TTPen: a high battery capacity rechargeable with universal power cable; **(B)** Levothyroxine pen-like dispenser, containing L-T4 vial and Bluetooth^©^ connection system that interfaces with smartphones.

By combining the need of target therapy for hypothyroid patients and the cutting-edge technology, our dream is represented by a compact, portable, automated, digital device able to administer to each patients the proper dosage of L-T4, namely TTPen (**Figure 1**). Try to think having a TTPen that is able to administer the perfect dosage needed.

TTPen consists of a charger and a pen-like dispenser. The charger provides a high battery capacity, it can be recharged with universal power cable, and it recharges the dispenser daily. The pen-like dispenser consists of a slot carrying the L-T4 vial and the dispenser. Each vial contains the monthly amount of L-T4 for a hypothetic 80 kg patient; different vials dosages are available according to the requirement based on weight, from 1.6 mcg/kg to 2.1 mcg/kg.

TTPen is made up of a material able to maintain thermal stability when exposed to both hot and cold temperatures. This last property, together with small size, makes TTPen portable, comfortable, and versatile. The dispenser releases the correct dose once a day. TTPen interfaces via Bluetooth^©^ with TTApp, an application for smartphone available both for clinicians and patients, that allows users to regulate, plan, and analyze L-T4 therapy. On one side, patients can set administration times and TTApp reminds them to assume L-T4 at the right time, with a further alarm if left on. Moreover, TTApp gives patients the opportunity to record symptoms possibly related to over- or undertreatment, and to mark TSH values. On the other side, on the basis of clinical and biochemical data, clinicians can regulate daily dosage remotely, making adjustments in order to ensure the perfect L-T4 delivery to patients. In addition, clinicians can suggest the timing of TSH dosage and TTApp reminds patients to perform blood exams. TTSystem (TTPen and its device) is also fitted with an alarm system advising patients if any error occurs or if the vial is running out. Finally, the data recorded by TTApp are transferred to data repository available for clinicians, according to current privacy legislation.

Forward thinking, this innovative technology can be implemented and adapted for Hospital setting. In fact, each hypothyroid inpatient is on L-T4 therapy at different dosages, and in each Department countless different dosages and formulations need to be stocked. This involves space consumption, excessive waste of paper and plastic, and increased risk of expired medicine. A larger TTPen, with larger vials, and an adapted TTApp could circumvent all these problems. Clinicians and nurses can register each patient and its daily dosage in TTApp; every morning the right L-T4 therapy can be administered to each patient in a semi-automatized way that minimizes error risk.

For sure, this innovative system makes hypothyroid patients reach the perfect personalized therapy. Another benefit, no less important, is represented by eco-sustainability. In fact, no more bulky packages are needed, no more great amounts of paper and plastic get wasted because each vial lasts about one month. The impact of this saving is minimal considering a patient, but, if we think to the great amount of hypothyroid patients, we can play our part in saving our world. If we extend this system to Hospitals, the great saving sticks up, and this should not be underestimated in the face of the global warming phenomenon.

Indeed, several difficulties could be found in the development of this system. Even though the technology seems similar or easier than the insulin pump one, development process requires the application of engineering knowledge, considerable financial allocations and a long pathway for obtaining needed authorizations. Recognizing the importance of these innovative strategies by pharmaceutical companies is a key point. However, as it is well demonstrated for insulin pumps, health technologies are cost-effective (91): in front of a high upfront investment, several economic benefits can be obtained, in particular healthcare systems can display a reduction of direct and indirect costs. This innovative product, improving self-management and providing therapy remotely, could reduce outpatients access; in fact, patients on stable L-T4 therapy don’t need any additional periodical clinical evaluation. In addition, ensuring a better target achieving, a significant reduction of the number of TSH measurement could be shown in trial based economic evaluations (92).

TTSystem is a simple, effective, and eco-sustainable system that could represent the starting point of a revolution in hypothyroid therapy. The development of a product that incorporates design, quality, and enables personalized therapy is a dream. Pharmaceutical industry should realize how this could comprehensively change the field of hypothyroidism and undertake the development and commercialization pathway overcoming the existing barriers hindering the adoption of Digital technologies.

## Conclusion

TTSystem represents the opportunity to cross a personalized L-T4 treatment over the new century. If it is the right way to tailored therapy, why not think of a similar system adapted to other medications, such as many psychiatric treatments or antibiotics?

In fact, antibiotic resistance, a global public health emerging crisis, has necessitated innovative solutions to counter the growing threat of untreatable bacterial infections. It has been attributed to the overuse and misuse of antibiotics, mainly due to incorrect dosage (93, 94). In this view, “precision antibiotics” or “personalized antibiotic therapy”, could offer a ray of hope in the battle against resistant bacterial strains (95).

**Figure 2:**
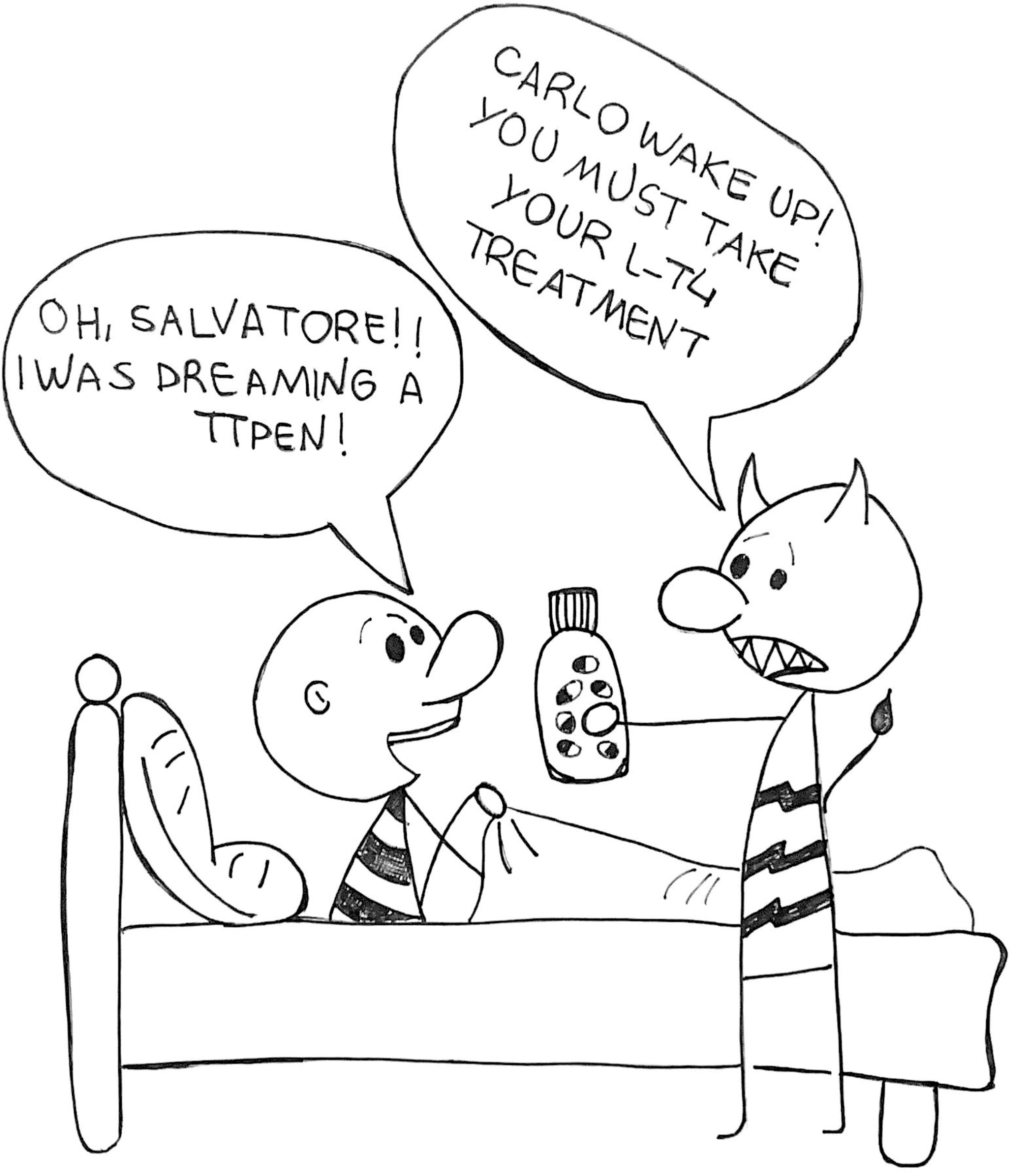
Carlo comes back to the present from his dream.

But let’s come back to the present… “Carlo wake up! You must take Your L-T4 treatment” “Oh, Salvatore, I was dreaming a TTPen” (**Figure 2**).

## Data availability statement

The original contributions presented in the study are included in the article/supplementary material. Further inquiries can be directed to the corresponding authors.

## Author contributions

CC: Conceptualization, Writing – review & editing. EG: Writing – original draft. SI: Conceptualization, Writing – review & editing.
